# Quantitative Assessment of Arthritis Activity in Rheumatoid Arthritis Patients Using [^11^C]DPA-713 Positron Emission Tomography

**DOI:** 10.3390/ijms21093137

**Published:** 2020-04-29

**Authors:** Maqsood Yaqub, Nicki J.F. Verweij, Simone Pieplenbosch, Ronald Boellaard, Adriaan A. Lammertsma, Conny J. van der Laken

**Affiliations:** 1 Amsterdam UMC—location VUmc, Radiology and Nuclear Medicine, De Boelelaan 1117, 1081 HV Amsterdam, The Netherlands; maqsood.yaqub@amsterdamumc.nl (M.Y.); s.pieplenbosch@amsterdamumc.nl (S.P.); r.boellaard@amsterdamumc.nl (R.B.); aa.lammertsma@amsterdamumc.nl (A.A.L.); 2 Amsterdam UMC—location VUmc, Amsterdam Rheumatology and immunology Center (ARC), De Boelelaan 1117, 1081 HV Amsterdam, The Netherlands; n.verweij@amsterdamumc.nl

**Keywords:** PET, quantification, simplified methods, pharmacokinetics, rheumatoid arthritis

## Abstract

Treatment for rheumatoid arthritis (RA) should be started as early as possible to prevent destruction of bone and cartilage in affected joints. A new diagnostic tool for both early diagnosis and therapy monitoring would be valuable to reduce permanent joint damage. Positron emission tomography (PET) imaging of macrophages is a previously demonstrated non-invasive means to visualize (sub)clinical arthritis in RA patients. We developed a kinetic model to quantify uptake of the macrophage tracer [^11^C]DPA-713 (*N*,*N*-diethyl-2-[2-(4-methoxyphenyl)-5,7-dimethylpyrazolo [1,5-a]pyrimidin-3-yl]acetamide) in arthritic joints of RA patients and to assess the performance of several simplified methods. Dynamic [^11^C]DPA-713 scans of 60 min with both arterial and venous blood sampling were performed in five patients with clinically active disease. [^11^C]DPA-713 showed enhanced uptake in affected joints of RA patients, with tracer uptake levels corresponding to clinical presence and severity of arthritis. The optimal quantitative model for assessment of [^11^C]DPA-713 uptake was the irreversible two tissue compartment model (2T3k). Both K_i_ and standardized uptake value (SUV) correlated with the presence of arthritis in RA patients. Using SUV as an outcome measure allows for a simplified static imaging protocol that can be used in larger cohorts.

## 1. Introduction

Rheumatoid arthritis (RA) is a systemic connective tissue disease that affects approximately 0.5% of adults in developed countries [1]. The chronic inflammation typical of RA may cause destruction of bone and cartilage in affected joints, leading to a decline in physical function and quality of life [2]. Recent international guidelines stress the importance of starting effective treatment as early as possible [3]. Therefore, a diagnostic tool for early diagnosis and therapy monitoring could be useful for reducing permanent physical damage.

Positron emission tomography (PET) is a non-invasive imaging technique with high sensitivity [4,5], that has shown high potential for visualizing RA disease activity [6]. The macrophage has been shown to be a specific target for RA PET imaging, because of its infiltration in the synovium of RA patients throughout the course of the disease and its reflection of treatment efficacy [7,8,9,10,11]. Previously, the macrophage tracer (*R*)-[^11^C]PK11195 (1-(2-chlorophenyl)-N-methyl-N(1-methyl-propyl)-3-isoquinoline carboxamide) has been used to image RA disease activity [12]. This tracer binds with nanomolar affinity to the translocator protein (TSPO), which is mainly found on (activated) macrophages and monocytes [13,14,15,16]. Although (*R*)-[^11^C]PK11195 could effectively visualize clinical arthritis in RA patients, identification of more subtle arthritis was often difficult due to the relatively high level of background uptake of (*R*)-[^11^C]PK11195 in peri-articular tissues [15].

High nonspecific binding and a low signal-to-noise ratio in brain studies of activated microglia has led to the development of a new generation of TSPO tracers, such as [^11^C]DPA-713 (*N*,*N*-diethyl-2-[2-(4-methoxyphenyl)-5,7-dimethylpyrazolo [1,5-a]pyrimidin-3-yl]acetamide), with a lower level of background uptake in the brain [17,18]. In subsequent pre-clinical and clinical proof of concept studies, it has been shown that [^11^C]DPA-713 is a promising novel candidate macrophage tracer for imaging arthritis activity in RA [12], as it provides higher absolute uptake in arthritic joints and higher target-to-background ratios as compared with (*R*)-[^11^C]PK11195 [19].

However, for this tracer, the optimal pharmacokinetic model for quantifying uptake of [^11^C]DPA-713 in RA patients still needs to be identified. Therefore, the primary aim of the present study was to develop a tracer kinetic arterial input model for describing [^11^C]DPA-713 uptake in arthritic joints of RA patients and to use this model to assess the performance of several simplified methods, such as the standardized uptake value (SUV).

## 2. Results

### 2.1. Clinical Data

Five patients with clinically active RA were included. Baseline patient demographics together with clinical and functional characteristics are summarized Table 1. The number of swollen joints per patient ranged from 5 to 12. Metatarsophalangeal joint (MTP) joints were most often affected (33%), followed by metacarpophalangeal (MCP) joints (26%), proximal interphalangeal (PIP) joints (21%), wrists (10%), and knees (10%).

**Figure 1:**
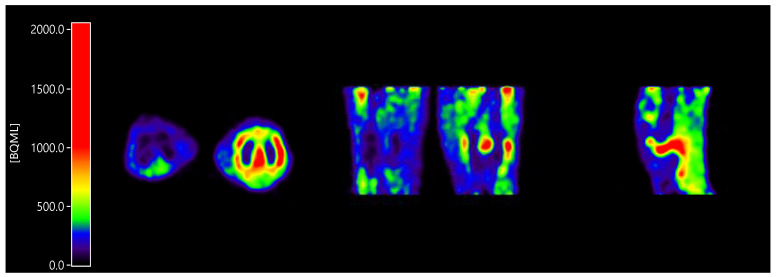
Enhanced [^11^C]DPA-713 (*N*,*N*-diethyl-2-[2-(4-methoxyphenyl)-5,7-dimethylpyrazolo [1,5-a]pyrimidin-3-yl]acetamide) uptake in a clinically inflamed knee joint compared to a clinically non-affected knee joint, in axial orientation (inflamed joint right), coronal orientation (inflamed joint right), and sagittal orientation (only inflamed joint).

All patients showed enhanced [^11^C]DPA-713 uptake in the clinically most affected joint within the field of view (Figure 1). Furthermore, in case of investigation of multiple joints within the field of view (e.g., hands), often more than one joint was present.

### 2.2. Input Functions for Dynamic PET Data Analyses

**Figure 2:**
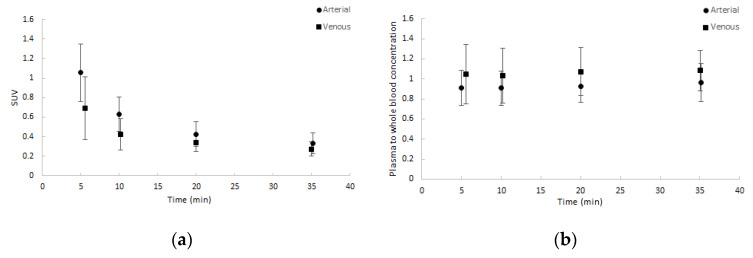
Average blood sample data (dots) with one SD range (error bars) as a function of time (minutes): (**a**) normalised whole blood standardized uptake value (SUV) and (**b**) plasma to whole blood concentration ratios. Both arterial and venous samples are shown.

Whole blood activity normalised to injected dose and patient weight showed good agreement amongst patients and between arterial and venous samples. Similar results were seen for the plasma to whole blood activity ratios. Both are depicted in Figure 2. Notably, the ratio of plasma to whole blood activity remained approximately 1, corresponding with a binding of approximately 55% in arterial samples and 65% in venous samples over time to blood cells.

**Figure 3:**
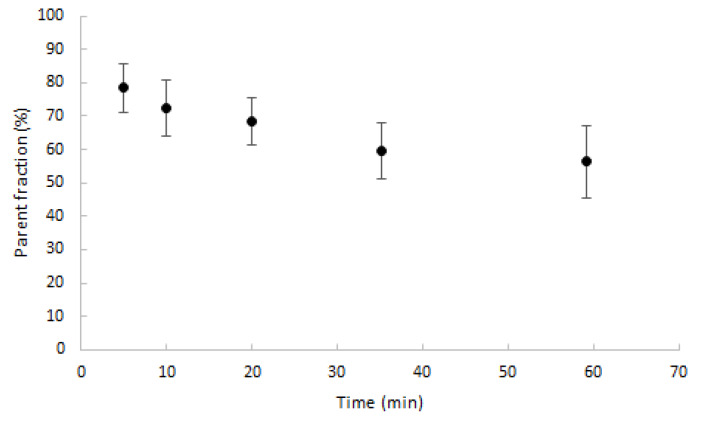
Average percentage of parent fractions in plasma (dots) with one SD range (error bars) as function of time (min).

Parent fractions decreased to, on average, 70% after 20 min post injection (min p.i.) and slowly decreased further thereafter, as shown in Figure 3. The maximum percentage of metabolites was 43.7%, measured after 60 min.

### 2.3. Kinetic Analyses Using Non-linear Plasma Input Models

To avoid non-convergence, it was necessary to implement boundaries for the kinetic parameters (Table 2). In particular, the lower limit for k_2_ improved overall determination of all kinetic parameters.

Visual assessment showed acceptable fits for all models in all segmented regions, except for the single tissue compartment model (1T2k) model in case of bone marrow time activity curves (TACs). Model preferences according to the Akaike information criterion are given in Table 3, showing that for all regions the irreversible two tissue compartment model (2T3k) model was the preferred model. Where the differences in the Akaike values between the reversible two tissue compartment model (2T4k) and 2T3k models were relatively small (≈2.4%), the difference between the 1T2k and 2T3k models were much larger (≈9.7%). Only a few RA affected regions showed preference for the 2T4k model.

**Figure 4:**
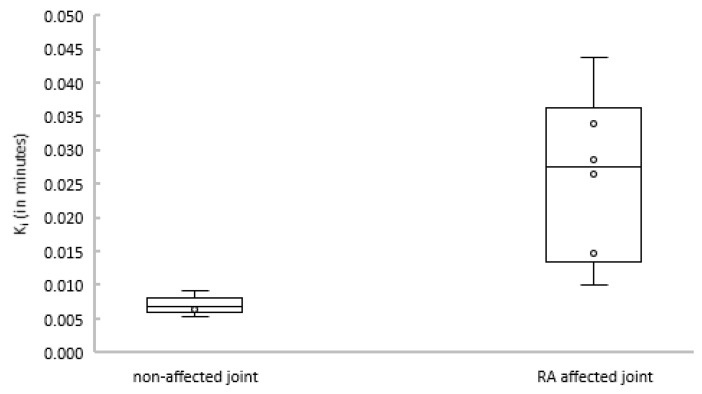
Boxplot depicting Ki estimates for non-affected (left) and rheumatoid arthritis (RA)-affected (right) joints.

Subsequently, the net influx constant K_i_ of the 2T3k model was compared between joints with and without clinical arthritis activity. Figure 4 shows that K_i_ was significantly different between affected and non-affected joints (*p* = 0.004).

### 2.4. Linearized and Simplified Methods

**Figure 5:**
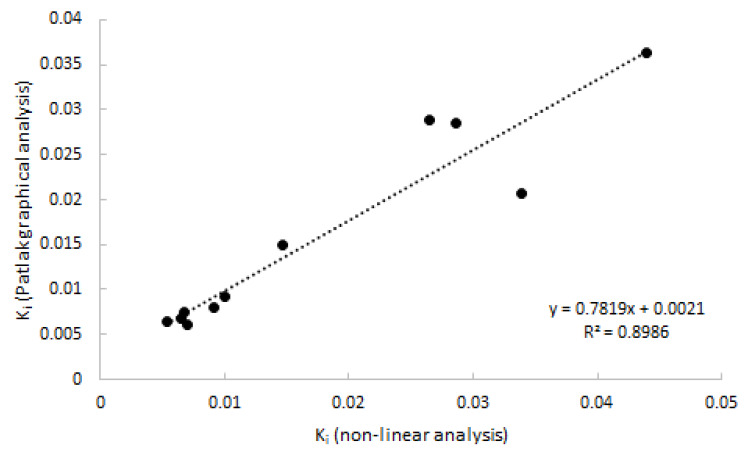
Correlation of K_i_ estimated using Patlak graphical analysis vs. K_i_ estimated using non-linear analysis. Data consist of both clinically affected and non-affected joints.

As 2T3K was determined to be the optimal model, Logan analysis was not performed. Patlak provided good linear fits from a starting time of 20 min p.i. onwards, which was shown to be superior to a starting time of 30 and 40 min p.i. Good agreement (*R^2^* = 0.90) was observed between Patlak-derived K_i_ and with 2T3k-derived K_i_ (Figure 5).

**Figure 6:**
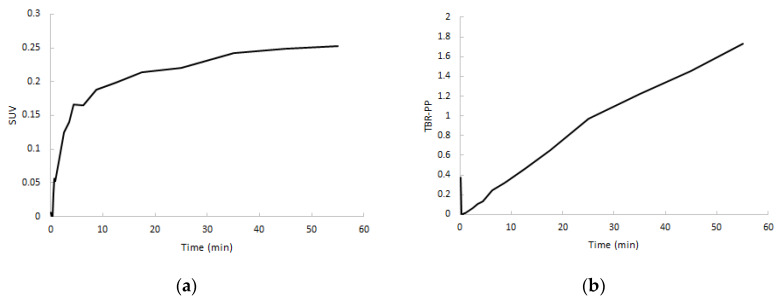
Normalized SUV curves (**a**) and target-to-metabolite corrected plasma ratio (TBR-PP) curves (**b**) of RA-affected joints from the same subject.

After a rapid initial uptake, SUV TACs showed a flattening in increase of uptake after approximately 5 min in both clinically non-affected and affected joints, whereas target-to-metabolite corrected plasma ratio (TBR-PP) demonstrated a consistent increase in uptake up to 60 min p.i. (Figure 6). TBR-PP and target-to-whole blood ratio (TBR-WB) curves showed a strong linear increase in all regions, and therefore no optimal interval could be defined.

**Figure 7:**
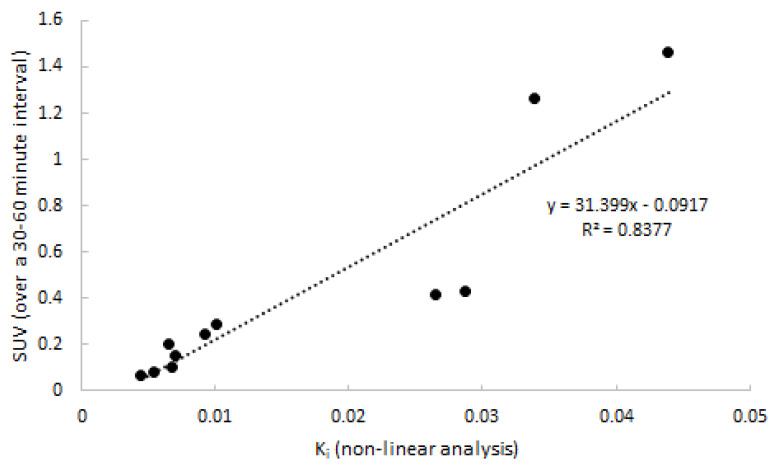
Correlation between SUV and 2T3k-derived K_i_. Data consist of both clinically affected and non-affected joints.

Next, quantitative accuracy of SUV was assessed. SUV over four intervals (20–40, 30–50, 30–50, and 40–60 min p.i.) was compared to 2T3k-derived K_i_. The SUV interval of 30–60 min p.i. showed the best correlation to K_i_, and is depicted in Figure 7 (*R*^2^ = 0.82). Two points distant from the correlation line are from the same subject and might have been due to inaccurate recording of patient weight. Differences between clinically affected and non-affected joints were significant (*p* = 0.018).

### 2.5. SUV in Relation to Clinical Activity

After establishing quantitative accuracy of SUV (30–60 min p.i.), the relation to clinical activity was assessed. SUV values and 2T3k-derived K_i_ values for both clinically arthritic and non-affected, contralateral joints were compared to clinical activity, as shown in Table 4.

At a patient level, neither K_i_ nor SUV showed a significant association with patient level-related clinical factors such as disease activity score of 44 joints (DAS44), number of swollen and/or tender joints, and serological parameters (C-reactive protein (CRP) and erythrocyte sedimentation rate (ESR)). In contrast, at a joint level, the severity of arthritis activity corresponded with SUV_peak_—the clinically most fulminant arthritis in the depicted joint (patients 3, 4, and 5) showed the highest SUV_peak_ values, whereas more chronic, latent arthritis (patient 1) showed a low SUV_peak_. The uptake in the clinically non-affected joint was always lower than the uptake in the affected joint within the same patient, but the uptake level varied remarkably between patients. No relationship was observed between tracer uptake in arthritic joints and medication use or dosage.

## 3. Discussion

In the present study, a kinetic model for [^11^C]DPA-713 uptake in arthritic joints of clinically active RA patients was developed using a metabolite-corrected arterial input function in combination with dynamic imaging. All patients showed enhanced [^11^C]DPA-713 uptake in clinically inflamed joints compared with clinically non-affected joints. Blood data showed good agreement among subjects and between arterial and venous sampling. The irreversible two-tissue compartment model proved to be the optimal quantitative model for assessing the rate of [^11^C]DPA-713 uptake, and K_i_ was able to significantly differentiate between clinically affected and non-affect joints. Furthermore, K_i_ and SUV (30–60 min p.i.) showed similar properties and high correlation, demonstrating the validity of SUV as a simplified method for whole-body [^11^C]DPA-713 studies. A correlation of SUV with several clinical factors was found, and the SUV appeared to be highly susceptible to degrees of (sub)clinical inflammation. As well as this, SUV can be used between groups, allowing for simplified static scanning protocols. These characteristics make [^11^C]DPA-713 a promising tracer for application in future studies in larger cohorts of RA patients.

A significant relationship was observed between clinical severity of arthritis and SUV at joint level. At patient level, it was found that patients with clinically very active arthritis (severe swelling and pain at physical examination) showed the highest SUV values, whereas the patient with smouldering and less distinguished arthritis showed low uptake. In addition, the increased SUV in the contralateral, non-affected joint of patient may point to depiction of subclinical arthritis because this joint had been painful for a longer time (without clinical swelling). This is in line with our previous findings, which showed that TSPO tracers including [^11^C]DPA-713 and [^11^C]PK11195 could possibly detect subclinical inflammation, which can precede development of clinical assessable arthritis later in time [19,20]. The addition of a control group of healthy volunteers in future [^11^C]DPA713 studies could further provide information about the range of uptake in non-inflamed joints.

Large differences in tracer uptake were found between investigated regions of joints. Although this can, at least in part, be attributed to clinical differences, regional differences in volume of interest (VOI) definition and position may also play a role. VOI size and location for joint uptake was determined by the type of affected joint (small vs. large). In a small VOI, for instance drawn on a small hand joint, assessed tracer uptake may be more affected by the limited spatial resolution than in a larger VOI drawn on a large joint. This affects both clinically affected and non-affected joints. Furthermore, size and positioning of the VOIs in blood pool regions also differed—some of which were drawn in the aorta descendens, but for one patient a small vessel in the foot had to be used. Future research should evaluate whether tracer kinetics are region-dependent.

Whereas both K_i_ and SUV showed the ability to differentiate between patients, as well as between clinically affected and non-affected joints in patients, their use for therapy monitoring has not yet been proven. Therapy given for RA can alter perfusion in the joints [21], which can incorrectly alter K_i_ and SUV values. This could cause misrepresentation of the actual macrophage activity. Therefore, further investigation of this tracer in a therapy monitoring setting is warranted.

Target binding did not appear to be influenced by metabolism or binding to other cells. [^11^C]DPA-713 binding to blood cells was determined as approximately 60% over time. As a detailed analysis of the binding in blood was not performed, specification of binding and the blood cells remain unknown. However, as TSPO tracers are known to bind to (activated) macrophages in arthritic joints [13,14,15], it may well be that [11C]DPA-713 binds (partly) to monocytes in the blood compartment, which migrate to the tissue and become activated macrophages, and/or binds to the macrophages that are already present in tissue. Although possible influence of metabolites on binding of tracer to target cannot be ruled out indefinitely, it is very implausible. The metabolism we measured was relatively slow and comparable to that of established TSPO tracer *(R)*-[^11^C]PK11195 [22]. As the pharmacokinetic model using metabolite corrected plasma input function was well capable of fitting and describing the target time activity curves, a significant effect on target binding by radiolabeled metabolites is unlikely.

Both visual assessment and Akaike information criteria showed an overall strong preference for the irreversible two tissue plasma input model in all assessed regions. However, the actual differences in the Akaike values between the models 2T4k and 2T3k were relatively small (≈2.4%), whereas the differences in Akaike values between the 1T2k and 2T3k were much larger (≈9.7%). Due to the slow kinetics of the tracer in uptake of joints, the optimal model within the used scan time is the 2T3k, and no significant tracer is released from the bound compartment (k_4_: ≈0.01 1/min). Notably, arthritic regions with preference for the reversible two-tissue model showed the highest uptake. K_i_ estimated using the irreversible two-tissue model showed a clear group separation between non-affected and arthritis regions. Therefore, 2T3k-based K_i_ can be used as a valid parameter for discrimination between non-affected and affected regions.

As expected from the analysis using non-linear models, only linearization according to Patlak showed acceptable fits. Patlak-derived K_i_ showed a good correlation with non-linear estimated K_i_ (*R^2^* = 0.83). When selecting the optimal simplified approach on the basis of a static scan interval, it is important that the uptake using that simplified parameter correlates well with K_i_. Additionally, the approach should show a plateau uptake as function of time, making the measure independent of both an input function and small changes in acquisition time of the static interval. Analysing SUV or TBR curves only showed a plateau in uptake in the case of SUV. Neither TBR normalised using blood samples nor TBR normalised using metabolite corrected plasma samples provided a plateau within the entire scan interval, as clearance of the (parent) tracer from blood was always substantially faster than clearance from tissue to blood. Therefore, in clinical practice, SUV should be a more reliable parameter outcome measure. High correlation was found and, similar to K_i_, a significant separation in uptake between clinically non-affected and clinically affected regions was seen.

The group size of the current study was small, with only five patients, making the assessment of correlations at a patient level difficult. Nevertheless, the number of non-affected and clinically arthritic regions was sufficient for proper kinetic modelling, especially given the clear preference of the irreversible two-tissue model. Interestingly, statistical analyses of linearized and simplified methods provided several significant results (when using *p* ≤ 0.05). Therefore, the sample size appears large enough to define optimal quantitative and simplified models.

## 4. Materials and Methods

### 4.1. Patients

In the period June to December 2017, five patients (1 male, age 58 ± 5 years) were included. Patients were eligible for inclusion if they had been diagnosed with RA according to the criteria of the American College of Rheumatology/European League Against Rheumatism [23], were at least 18 years of age, received stable treatment (non-steroidal anti-inflammatory drugs for at least 1 month, or disease-modifying anti rheumatic drugs and/or biologicals for at least 3 months), and received a maximum of 10 mg of oral corticosteroids per day. In addition, patients had to have clinical arthritis, defined as swelling in the joint as assessed by a physician, in at least one hand, knee, or foot joint. Exclusion criteria were previous exposure to research-related radioactivity above 5 mSv in the previous year, the use of experimental drugs in the previous 3 months, pregnancy and/or breastfeeding, anaemia (haemoglobin < 6.0 mmol/L), or renal insufficiency (GFR < 30 mL/min/1.73 m^2^).

### 4.2. Clinical Assessment

Demographical and clinical data were obtained after inclusion. Disease activity was assessed through physical examination of 44 joints [24], patient questionnaires, and the measurement of inflammatory markers such as C-reactive protein (CRP) and erythrocyte sedimentation rate (ESR) in blood. All patients gave written informed consent prior to inclusion in the study, which was approved by the Medical Ethics Review Committee of the Amsterdam University Medical Center, located in the VU University Medical Center (Project code: 2016.547, approval date: 24 February 2017).

### 4.3. Scanning Protocol

Each subject was scanned on an Ingenuity TF PET/CT scanner (Philips Medical Systems, Best, the Netherlands). The field of view (18.4 cm axial) was placed around the clinically most inflamed joint, as determined by a physician (N.V.). The scan protocol consisted of a low-dose CT for attenuation correction and anatomical positioning, followed by intravenous injection of 406 ± 5 MBq [^11^C]DPA-713 at the start of a dynamic PET scan of 60 min. Directly after dynamic scanning was finished, a static scan of 4 min was performed of the same region in order to assess the relationship between dynamic and static images.

Acquired list mode data were reconstructed into 19 frames (1 × 15, 3 × 5, 3 × 10, 4 × 60, 2 × 150, 2 × 300, and 4 × 600 seconds). Data were reconstructed using 3D RAMLA in combination with CT-based attenuation correction, providing images with a final voxel size of 4 × 4 × 4 mm^3^ and a spatial resolution of 5 mm full width at half maximum (FWHM). Reconstructions included all usual corrections, such as detector normalization, and decay, dead time, attenuation, randoms, and scatter corrections.

The protocol also included continuous arterial sampling, starting 2 min prior to tracer injection and continuing for the first 25 min of the dynamic PET scan, using a dedicated on-line detection system [25]. In addition, at 5, 10, 20, 35, and 60 min post-injection (p.i.), discrete arterial samples were taken from the arterial line, requiring arterial sampling to be interrupted briefly at 5, 10, and 20 min post-injection. After each sample, the arterial line was flushed with heparinised saline in order to avoid clotting of blood. In addition, venous samples were withdrawn from an intravenous catheter placed on the contralateral side of the tracer infusion at the same times as arterial samples, with an additional sample at 70 min post-injection. Input curves were generated following a step-wise procedure: (1) the continuous arterial sampling curve was corrected for flushing periods; (2) the continuous arterial data were calibrated using the discrete arterial samples and extrapolated using a multi-exponential fit to the late discrete samples (>25 min p.i.); (3) the data were corrected for plasma to whole blood ratios (measured from the samples); (4) the data were corrected for metabolites measured in plasma of discrete blood samples per previously described protocol [26]; and (5) the data were corrected for delay, relative to the PET scan, finally resulting in a metabolite-corrected arterial plasma input curve.

### 4.4. Image Segmentation

Using the low dose CT images, volumes of interest (VOIs) were drawn manually within the boundaries of clinically active joints (knee, MCP joint, MTP joint, and wrist), avoiding blood vessels as much as possible. In addition, VOIs were also drawn on contralateral, clinically non-affected joints (to determine background uptake), bone marrow, and blood pool (largest artery in the field of view) regions. Subsequently, these VOIs were projected onto the corresponding dynamic [^11^C]DPA-713 images in order to extract regional time activity curves (TACs). Finally, for clinical assessment of static PET scans performed at approximately 60 to 64 min p.i. only, SUV_peak_-based regions were defined for the target lesions, that is, automatic placement of a 1 mL spherical VOI on the lesion so as to obtain the highest mean SUV value for the sphere.

### 4.5. Kinetic Analysis Using Non-linear Plasma Input Models

TACs were analysed using the three conventional compartmental models, all requiring a metabolite-corrected arterial plasma input function. All compartment models included an additional parameter for fractional blood volume (*V*_B_) and consisted of a single tissue compartment model (1T2k), irreversible two tissue compartment model (2T3k), and reversible two tissue compartment model (2T4k).

Selection of the optimal model was determined by number of outliers, visual fit quality, and Akaike information criterion [27]. The optimal fitting model was used to derive various model-dependent kinetic parameters such as influx rate constants (K_1_, k_2_, k_3_, and K_i_), binding potential (*BP*_ND_), and/or volume of distribution (*V*_T_). Parameter estimates from these models were assessed for their ability to identify differences between RA-affected and non-affected regions.

### 4.6. Linearized and Simplified (Static) Analysis

The accuracy and precision of linearized plasma input methods (either Patlak or Logan, t* > 20, 30, and 40 min p.i.) and several simplified static approaches were evaluated by comparing outcome measures with corresponding kinetic parameters obtained with the optimal non-linear compartment model. Simplified static approaches consisted of measures of regional average tracer uptake over an interval (20–40, 30–50, 30–60, and 40–60 min p.i.) normalised to (a) patient weight and injected dose (SUV), (b) activity in arterial whole blood (target-to-whole blood ratio (TBR-WB)), and (c) metabolite-corrected plasma (target-to-metabolite corrected plasma ratio (TBR-PP)). The optimal SUV interval was defined as the interval for which uptake reached a plateau and showed best correlation to the kinetic parameter derived from the optimal non-linear (NLR) method.

### 4.7. Statistical Analysis

Continuous variables with Gaussian distribution were summarized as mean ± standard deviation (SD) and 95% confidence intervals (CI). Non-normally distributed variables were summarized as median and interquartile range (IQR). SPSS version 22.0 software (SPSS, Chicago, IL, USA) was used to assess the distribution of both clinical and PET data.

To assess the correlation of dynamic data with simplified static measures, the Pearson correlation coefficient (*R^2^*) was used. In addition, dynamically derived K_i_ of arthritic joints and non-affected joints were compared using the unpaired two-tailed *t*-test (*p). p*-values smaller than 0.05 were considered as statistically significant.

## 5. Conclusions

[^11^C]DPA-713 demonstrated enhanced uptake in clinically active joints of RA patients. Several kinetic models were fitted to the data, and the irreversible two-tissue compartment model proved to be the optimal quantitative model for assessing the rate of [^11^C]DPA-713 uptake. K_i_ was able to distinguish between different levels of RA activity. Furthermore, SUV showed similar properties to K_i_, and therefore appears to be a valid simplified method for whole-body [^11^C]DPA-713 studies. A correlation of SUV with clinical arthritis activity at joint level was found, and data suggest that SUV is also highly susceptible to degrees of (sub)clinical inflammation. Lastly, SUV can be used between groups, allowing for simplified static scanning protocols. These characteristics make [^11^C]DPA-713 a promising tracer for application in future studies in larger cohorts of RA patients.

## Figures and Tables

**Table 1:** Baseline patient demographics with clinical and functional characteristics.

Baseline Patient Demographics	
Male, number (%)	1 (20)
Age, years (mean ± SD)	58 ± 5
Height, cm (mean ± SD)	169 ± 15
Weight, kg (mean ± SD)	82 ± 16
Disease duration, months (mean ± SD)	314 ± 195
Anti-CCP positivity, number (%)	3 (60)
IgM RF positivity, number (%)	3 (60)
DAS 44 (mean ± SD)	2.78 ± 0.43
44-swollen joint count (median ± IQR)	7 ± 5
44-tender joint count (median ± IQR)	6 ± 7
CRP, mg/mL (median ± IQR)	3 ± 9
ESR, mm/h (median ± IQR)	9 ± 27
VAS disease activity, 0 – 100 mm (mean ± SD)	45 ± 19
DMARD therapy, number (%)	3 (60)
Oral prednisolone, number (%)	4 (80)
NSAID therapy, number (%)	2 (40)

Abbreviations: IgM RF: immunoglobulin M rheumatoid factor, DAS: disease activity scale, CRP: C-reactive protein, ESR: erythrocyte sedimentation rate, VAS: visual analogue scale, DMARD: disease modifying anti-rheumatic drug, NSAID: non-steroidal anti-inflammatory drug.

**Table 2:** Parameter boundaries used during non-linear regression analysis.

Kinetic Parameter	Lower Bound	Upper Bound
K_1_	0.005	0.2
k_2_	0.01	0.5
k_3_	0.01	1.0
V_T_	0.01	50
V_b_	0.001	1.0

**Table 3:** Preference for single tissue compartment model (1T2k), irreversible two tissue compartment model (2T3k), and reversible two tissue compartment model (2T4k) models, given in %, for various regions on the basis of the Akaike information criterion.

Volume of Interest	1T2k	2T4k	2T3k
Bone marrow	0	0	100%
Non-affected joint	0	0	100%
Affected joint	0	28.6%	71.4%

**Table 4:** Clinical activity with corresponding SUV_peak_ for arthritic and non-affected, contralateral joints.

Patient	Clinical Activity	Type of Joint	Arthritic Joint	Non-Affected, Contralateral Joint
1	+	Knee	3.6	2.4
2	++	MCP2	10.2	4.0
3	+++	Knee	16.5	2.4
4	+++	Knee	12.4	8.1
5	+++	MTP1	12.7	2.0
		Average	11.1	3.8
